# Aprepitant versus Olanzapine in Patients of Breast Cancer on Adriamycin and Cyclophosphamide Regimen – Role in Effectiveness of Prevention of Nausea and Vomiting

**DOI:** 10.15388/Amed.2025.32.1.22

**Published:** 2025-02-18

**Authors:** Indrani Devi Sarma, Sukainnya Buragohain, Joonmoni Lahon, Indrani Bhagawati, Neelakshi Mahanta, Dibyajyoti Saikia

**Affiliations:** 1 Department of Pharmacology, AIIMS Guwahati, Changsari, Assam, India; 2 Department of Pharmacology, Nalbari Medical College and Hospital, Assam, India; 3 Department of Medical Oncology, State Cancer Institute, Guwahati, Assam, India; 4 Department of Pharmacology, AIIMS Guwahati, Changsari, Assam, India

**Keywords:** Olanzapine, Aprepitant, Chemotherapy-Induced Nausea and Vomiting (CINV), Breast Cancer, Highly Emetogenic Chemotherapy (HEC), Antiemesis, olanzapinas, aprepitantas, chemoterapijos sukeltas pykinimas ir vėmimas (CINV), krūties vėžys, labai emetogeninė chemoterapija (HEC), antiemezė

## Abstract

**Background:**

Chemotherapy-induced nausea and vomiting (CINV) is a significant concern for patients undergoing highly emetogenic chemotherapy (HEC). This study compares the efficacy of aprepitant and olanzapine in preventing CINV in breast cancer patients receiving Adriamycin and Cyclophosphamide (AC).

**Methods:**

A prospective, comparative, observational study was conducted over one year at the State Cancer Institute, Guwahati, India. 103 chemotherapy-naïve breast cancer patients were enrolled and divided into two groups: aprepitant and olanzapine, both receiving standard therapy with ondansetron and dexamethasone. CINV outcomes were assessed using the Multinational Association of Supportive Care in Cancer (MASCC) Antiemesis Tool over five days post-chemotherapy. Acute (0–24 hours) and delayed (24–120 hours) nausea and vomiting were evaluated. Side effects were documented and compared between groups.

**Results:**

Olanzapine demonstrated significantly better control of acute nausea compared to aprepitant (*p* < 0.05). It also showed a trend towards superior efficacy in delayed nausea, though statistical significance was not reached. There was no significant difference between aprepitant and olanzapine in preventing acute or delayed vomiting. The olanzapine group experienced more frequent side effects, but the difference was statistically insignificant.

**Conclusion:**

Olanzapine exhibited greater efficacy in preventing nausea, particularly in the acute phase, compared to aprepitant. However, its higher side effect profile suggests that careful patient selection is necessary. Both agents remain effective options for CINV management, with olanzapine offering an advantage in nausea prevention.

## Abstract (it)

**Įvadas:**

Chemoterapijos sukeltas pykinimas ir vėmimas (CINV) yra didelė problema pacientėms, kurioms taikoma labai emetogeninė chemoterapija (HEC). Šiame tyrime lygintas aprepitanto ir olanzapino veiksmingumas siekiant išvengti CINV krūties vėžiu sergančioms pacientėms, kurioms skiriamas adriamycinas ir ciklofosfamidas (AC).

**Metodai:**

Valstybiniame vėžio institute, Gvahačio mieste, Indijoje, per vienerius metus buvo atliktas prospektyvinis, lyginamasis, stebimasis tyrimas. Į tyrimą buvo įtrauktos 103 chemoterapija negydytos krūties vėžiu sergančios pacientės, jos buvo suskirstytos į dvi grupes pagal joms skiriamą pykinimo ir vėmimo prevencijai vaistą: aprepitantą ir olanzapiną, abiejų grupių pacientėms buvo taikomas standartinis gydymas ondansetronu ir deksametazonu. CINV rezultatai buvo vertinami naudojant Daugianacionalinės vėžio palaikomojo gydymo asociacijos (MASCC) antiemetikus penkias dienas po chemoterapijos. Buvo vertinamas ūminis (0–24 val.) ir uždelstas (24–120 val.) pykinimas ir vėmimas. Šalutinis poveikis buvo dokumentuojamas ir lyginamas tarp grupių.

**Rezultatai:**

Olanzapinas daug labiau tiko geresnei ūminio pykinimo kontrolei nei aprepitantas (p < 0,05). Taip pat nustatyta, kad jis veiksmingesnis ir uždelsto pykinimo atveju, nors statistinio reikšmingumo nebuvo pasiekta. Reikšmingo skirtumo tarp aprepitanto ir olanzapino pagal galimybę užkirsti kelią ūminiam ar uždelstiniam vėmimui nebuvo. Olanzapino grupės pacientėms dažniau pasireiškė šalutinis poveikis, tačiau skirtumas buvo statistiškai nereikšmingas.

**Išvada:**

Olanzapino veiksmingumas užkirsti kelią pykinimui didesnis, ypač ūminėje fazėje, palyginti su aprepitantu. Tačiau didesnis jo šalutinio poveikio profilis rodo, kad būtina kruopščiai atrinkti pacientus. Abu vaistai yra veiksminga CINV gydymo priemonė, tačiau olanzapinas turi pranašumą pykinimo prevencijos srityje.

## Introduction

Breast cancer has always been a major health issue, especially in India, where it has become a primary reason for cancer and death among women [1,2]. It is one of the most common cancers afflicting women [3]. *Highly Emetogenic Chemotherapy* (HEC) regimens, like Adriamycin and Cyclophosphamide (AC), frequently result in severe side effects like nausea and vomiting [4]. Aprepitant, a neurokinin-1 (NK1) receptor antagonist, is one medication that has been approved to prevent nausea and vomiting caused by chemotherapy [5]. Olanzapine is atypical antipsychotic used ‘off-label’ to prevent CINV, and several studies have reported its efficacy [6]. Studies comparing these agents indicate that both can significantly reduce CINV, but further research is needed to examine their side effect profiles and the overall effectiveness to enhance treatment protocols for breast cancer patients receiving AC-based HEC. The primary objective of this study was to evaluate the effectiveness of Olanzapine and Aprepitant in preventing nausea. Vomiting and adverse effects of these two drugs in patients with breast cancer who were receiving AC-based HEC were taken as the secondary objective.

## Materials and Methods

### Study design and participants

This observational study spanned a year and was undertaken from May 2020 to April 2021 at the State Cancer Institute’s day-care centre in Gauhati Medical College, Guwahati, Assam. This prospective, comparative study included chemotherapy-naïve breast cancer patients aged 18 and above, scheduled to receive the *Highly Emetogenic Chemotherapy* (HEC) regimen of Adriamycin and Cyclophosphamide (AC), and provided informed consent. Ethical clearance was obtained from the Institutional Ethics Committees of both Gauhati Medical College and Hospital and the State Cancer Institute with the ethical approval letter Nos. MC/190/2007/Pt-11/Dec-2019/29 and SCI/ECR/2020/06, respectively.

Eligibility criteria also included a serum creatinine level of at least 2.0 mg/dL, AST and ALT levels not higher than three times the upper normal limit, an absolute neutrophil count of at least 1500/mm^3^, haemoglobin of 8 g/dL or greater, a platelet count of at least 1 x 10^5^/µL, a total leukocyte count of 4000/mm^3^ or more, normal electrolyte levels, and a left ventricular ejection fraction of 50% or above.

Patients were excluded if they reported nausea or vomiting before enrolment, and those receiving HEC regimens over multiple days of AC or other chemotherapy protocols were not included, either. The study also excluded individuals with severe cognitive impairment, central nervous system diseases (e.g., metastases to the brain, seizure disorders), and those treated with antipsychotics and opioids. Assessing the effectiveness of Olanzapine and Aprepitant in preventing nausea and vomiting in patients with breast cancer undergoing AC-based HEC was main objective of this study. It also did not include individuals with uncontrolled diabetes mellitus, pregnant or lactating women, severely debilitated patients, or those who refused to provide consent.

#### Procedures

The study employed the consecutive sampling method, utilizing a standardized case record form to gather data from participants. The *MASCC Antiemesis Tool* (MAT) was employed for the evaluation of CINV in the selected patients, which is free for use by non-commercial entities [7]. The patients were monitored for five days immediately following chemotherapy. The MAT was used to evaluate the occurrence and frequency of nausea and vomiting, with nausea intensity measured on a visual analog scale across a range from 0 to 10. Symptom severity was categorized by employing the *Common Terminology Criteria for Adverse Events* (CTCAE) version 5.0 (8) as mild (1 to 4), moderate (5 to 7), or severe (8 to 10) [9]. The patients were assessed after completing a single cycle of chemotherapy, with the option for rescue treatment based on clinical needs. The participants were split into two groups: one receiving Aprepitant, with a 125 mg capsule on day zero followed by 80mg daily on Days 2 and 3 post-chemotherapy, and the other group receiving Olanzapine, with a 10mg tablet on day zero followed by 10mg daily on Days 2, 3, and 4 post-chemotherapy. Additionally, on day 1, both groups received an 8mg intravenous dosage of Ondansetron and 8 mg intravenous Dexamethasone one hour before chemotherapy. On days 2, 3, and 4, they received oral Dexamethasone once daily.

#### Outcome measures

Primary outcome of occurrence of nausea in acute, delayed, and the overall phases were between the two groups. The outcome measures were recorded from day zero to day five. Incidence of vomiting was taken as secondary outcome, and it was recorded similarly.

The sample size was determined by using data from previous studies [10] which reported an incidence of the overall nausea rates of 69% with Olanzapine- and 38% with Aprepitant-based regimen, respectively. By using a two-sided test for comparing two proportions, with a significance level of 0.05, and a power of 90%, the required sample size came out to be 52 participants [11].

#### Statistical analysis

Descriptive analysis was used to compare the baseline characteristics of both groups. Continuous variables of age and the body surface area are presented as the mean and the standard deviation. Categorical variables of the menstrual status, comorbidities, TNM prognostic staging receptor positivity, and alcohol use history are presented as proportions and frequency. For comparisons of study outcomes, the odds ratio was calculated, and 0.05 was taken as the significance level. Analysis and visualization were done in the *R* software version 2024.04 for *Windows* (Windows NT 10.0; Win64; x64).

## Results

### Study participants

Out of 103 included patients, 52 received Aprepitant and 51 received Olanzapine. The baseline characteristics of the Aprepitant and Olanzapine arms show comparable demographic and clinical profiles. Overall, these baseline characteristics highlight that the two groups were broadly similar, with minor differences in disease staging and receptor status.

**Table 1:** Baseline characteristics of Aprepitant and Olanzapine groups

BASELINE CHARACTERISTICS	APREPITANT (n=52)	OLANZAPINE (n=51)	STATISTICAL TEST	P VALUE
*AGE*	46.2±8.1	46.1±8.5	T-TEST	0.95
*BSA*	1.5±0.1	1.5±0.1	T-TEST	1
*POSTMENOPAUSAL*	27	27	Chi-Square test	0.91
*H/O Alcohol intake*	4	3	Chi-Square test	0.72
*Comorbidities*	Absent (36) DM (1) HTN (9) DM + HTN (5) Others (1)	Absent (35) DM (1) HTN (8) DM + HTN (6) Others (1)	Chi-Square test	0.99
*ER positive*	26	23	Chi-Square test	0.62
*PR positive*	23	20	Chi-Square test	0.60
*Her2NEU*	3 positives	5 positives	Chi-Square test	0.30
	46 negatives	44 negatives		
	3 equivocal	2 equivocal		
*TNM*	I 11 II 9 III 20 IV 12	I 6 II 15 III 22 IV 8	Chi-Square test	0.28

#### Treatment outcomes

The results comparing the effects of the Aprepitant group and the Olanzapine group in preventing CINV highlight some key findings.

In acute nausea, a greater number of patients in the Aprepitant group (19 out of 52) experienced nausea compared to the Olanzapine group (9 out of 51). This difference was found to be significant. Similarly, in delayed nausea, patients who experienced this symptom were more numerous in the Aprepitant group (11) than in the Olanzapine group (4 patients), although the results are not statistically significant. Regarding delayed nausea, 11 patients in the Aprepitant group and 4 in the Olanzapine group experienced this side effect. Although this indicates a trend toward an increased nausea in the Aprepitant group, the difference is not statistically significant.

In the overall phase, nausea control was greater in the Olanzapine group as compared to the Aprepitant group. 8 patients in the Olanzapine group experienced nausea as opposed to 13 in the Aprepitant group. The difference between the groups, however, had no statistical significance.

For acute vomiting, 6 patients of 52 total in the Aprepitant group and 7 patients of 51 total in the Olanzapine group experienced this symptom, indicating that the difference between the two groups in reducing acute vomiting was insignificant. For delayed vomiting, 7 patients in the Aprepitant group and 3 in the Olanzapine group reported this side effect which shows a trend toward higher vomiting in the Aprepitant group, but these results are not statistically significant given the wide confidence intervals. Both groups showed similar efficacy with 7 patients of each group experiencing vomiting symptoms in the overall phase. No statistical significance was found between the groups in this regard.

#### Safety

Finally, the incidence of side effects was recorded, and the difference was statistically not significant, but lower rates of side effects were found in the Aprepitant group, with 2 patients affected compared to 6 in the Olanzapine group, thus indicating that side effects were less common in the Aprepitant group.

**Table 2:** Comparison table of Aprepitant and Olanzapine

	Aprepitant group N=52		Olanzapine group N=51		OR (CI)	RR (CI)
	Present	Absent	Present	Absent		
Acute nausea	19	33	8	43	2.6869* (1.0763 to 6.7074)	2.0705* (1.0357 to 4.1392)
Delayed nausea	11	41	4	47	3.1524 (0.9320 to 10.6633)	2.6971 (0.9185 to 7.9202)
Overall Nausea	16	36	8	43	2.3889 0.9172 to 6.2220	1.9615 0.9213 to 4.1764
Acute Vomiting	6	46	7	44	0.82 (0.2554 to 2.6315)	0.8407 (0.3032 to 2.3307)
Delayed vomiting	7	45	3	48	2.4889 (0.6062 to 10.2184)	2.2885 (0.6261 to 8.3652)
Overall Vomiting	7	45	7	44	1.0227 0.3313 to 3.1567	0.9808 0.3704 to 2.5972
Side Effects	2	50	6	45	0.3000 (0.0576 to 1.5625)	0.3269 (0.0692 to 1.5449)

OR = Odds Ratio, RR = Relative risk, CI = Confidence Interval, * = statistically significant.

## Discussion

In order to avoid CINV, this study compared Olanzapine to Aprepitant. It found that Olanzapine significantly reduced acute nausea, and also exhibited a discernible trend toward a reduction in delayed nausea. On the other hand, there was no discernible variation in effectiveness in terms of preventing vomiting. This was similar to the findings of other studies, such as Zhang et al. [12]. Aprepitant, an NK1 receptor antagonist, and Olanzapine, an atypical antipsychotic with antiemetic effects, act on distinct pathways involved in the emetic response [13]. These differences in their mechanisms of action may help explain the variations observed in their effectiveness for controlling nausea and vomiting.

In our study, Olanzapine was associated with 82% prevention of acute nausea. Other studies have reported that prevention of acute nausea by Olanzapine is 70–74% [14,15]. The difference between Olanzapine and Aprepitant groups in the case of acute phase nausea prevention was significant.

In acute vomiting, results between the two groups were found to be statistically insignificant. This aligns with previous studies that have suggested that both agents are effective in the acute phase of CINV management, and that there is no difference between their effect in the acute phase of CINV [16]. However, the more pronounced difference in delayed vomiting, where Olanzapine demonstrated a lower incidence (5.9% vs. 13.5% for Aprepitant), indicates a potential trend favouring Olanzapine, although the results did not reach statistical significance levels. Similar results of a positive trend for Olanzapine were also reported in other studies [17]. This finding is consistent with literature suggesting that Olanzapine may be more effective in managing delayed emesis due to its broader receptor activity, including dopamine and serotonin pathways [18].

The results for acute nausea were significant, with Olanzapine showing a lower incidence compared to Aprepitant. Additionally, this conclusion is consistent with data that has already been published [19]. Olanzapine may also be effective in treating delayed nausea, as shown by the tendency (7.8% for Olanzapine vs. 21.1% for Aprepitant), even though the results were found to be not statistically significant.

Despite the efficacy of Olanzapine, the higher incidence of side effects (11.8% for Olanzapine vs. 3.8% for Aprepitant) raises important considerations for clinical practice. Similar concerns were expressed by other studies where they stated that treatment with Olanzapine resulted in more side effects than the other drug regimen, with somnolence being a major one [20]. The side effects associated with Olanzapine, while not statistically significant, may be a concern for patients who are particularly sensitive to antipsychotic medications [21]. Careful selection of patients and their monitoring when using Olanzapine as part of an antiemetic regimen may be required as seen in other systematic reviews [22,23].

Olanzapine appears to offer significantly greater protection against delayed nausea compared to Aprepitant, especially in later cycles of chemotherapy. Unlike vomiting – which is primarily controlled by the *Chemoreceptor Trigger Zone* (CTZ) – nausea is influenced by a broader network of brain centers, including those involved in psychological and anticipatory responses. This means that once patients have experienced chemotherapy-induced nausea and vomiting (CINV) in earlier cycles, they may become more susceptible to future episodes, and a drug with a multi-modal mechanism like Olanzapine could therefore be particularly beneficial.

Given its diverse actions, Olanzapine might serve as a more effective prophylactic antiemetic over multiple chemotherapy cycles. However, it is important to note that current literature does not include any randomized controlled trials specifically addressing its efficacy in later cycles. Further studies are still needed to confirm these observations.

## Limitations

Our study had a number of limitations. Owing to the small sample size, the power of the study was low. Due to the observational nature of the study design, no randomization or blinding was done, thus leaving a potential for bias. However, the baseline characteristics did not have any statistically significant difference. The study considered only one dosing schedule. The patients were followed-up for a short period of time post chemotherapy.

## Conclusions

The results imply that, in comparison to Aprepitant, Olanzapine may offer superior protection against both acute and delayed nausea. The comparable efficacy in acute vomiting indicates that both agents are viable options for CINV management. However, the higher side effect profile associated with Olanzapine necessitates further investigation and consideration in clinical decision-making. Future studies with larger sample sizes could help to clarify these trends and inform best practices in the prevention of CINV.
